# Evaluation of Outcome after Total Hip Arthroplasty for Femoral Neck Fracture: Which Factors Are Relevant for Better Results?

**DOI:** 10.3390/jcm13071849

**Published:** 2024-03-23

**Authors:** Paolo Schiavi, Francesco Pogliacomi, Matteo Bergamaschi, Francesco Ceccarelli, Enrico Vaienti

**Affiliations:** Orthopedic Clinic, Department of Medicine and Surgery, University Hospital of Parma, 43126 Parma, Italy; francesco.pogliacomi@unipr.it (F.P.); matteo.bergamaschi1@studenti.unipr.it (M.B.); francesco.ceccarelli@unipr.it (F.C.); enrico.vaienti@unipr.it (E.V.)

**Keywords:** femoral neck fracture, total hip arthroplasty, direct anterior approach, outcome

## Abstract

**Background:** Femoral neck fractures (FNFs) are frequent orthopedic injuries in elderly patients. Despite improvements in clinical monitoring and advances in surgical procedures, 1-year mortality remains between 15% and 30%. The aim of this study is to identify variables that lead to better outcomes in patients treated with total hip arthroplasty (THA) for FNFs. **Methods:** All patients who underwent cementless THA for FNF from January 2018 to December 2022 were identified. Patients aged more than 80 years old and with other post-traumatic lesions were excluded. Patient data and demographic characteristics were collected. The following data were also registered: time trauma/surgery, surgical approach, operative time, intraoperative complications, surgeon arthroplasty-trained or not, and anesthesia type. In order to search for any predictive factors of better short- and long-term outcomes, we performed different logistic regression analyses. **Results:** A total of 92 patients were included. From multivariable logistic regression models, we derived that a direct anterior surgical approach and an American Society of Anesthesiologists (ASA) classification < 3 can predict improved short-term outcomes. Moreover, THAs performed by surgeons with specific training in arthroplasty have a lower probability of revision at 1 year. Mortality at 1 year was ultimately influenced by the ASA classification. **Conclusions:** A direct anterior approach and specific arthroplasty training of the surgeon appear to be able to improve the short- and long-term follow-up of THA after FNF.

## 1. Introduction

Femoral neck fractures (FNFs) are very common injuries in the elderly population. Recent studies have reported that the prevalence of FNFs is expected to show a strong increase worldwide by 2035 to 2050 [1,2,3,4,5,6].

Hip fractures have an important impact on the US healthcare system, with an annual incidence of 1000 per 100,000 beneficiaries [7,8]. It is estimated that the incidence of FNFs will increase from 1.66 million in 1990 to 6.26 million by 2050 [9]. In terms of type of treatment, the anatomic site of fracture plays an important role in determining the procedure needed. Intracapsular injuries consist of subcapital and transcervical groups, with arthroplasty being the common surgical treatment. In contrast, extracapsular fractures occur distal to the joint capsule insertion and can be distinguished into basicervical, intertrochanteric, and pertrochanteric groups, with osteosynthesis being the best recognized treatment [9]. 

Despite improvements in clinical monitoring and advances in surgical treatment, the 1-year mortality rates in patients who reported an FNF remain considerable, being 15% to 30% [7,10].

Currently, the common treatment of FNFs is internal fixation in 60% of cases, hemiarthroplasty in 30% of cases, and total hip arthroplasty (THA) in the remaining percentage of cases [11,12].

However, we have to consider that the use of THA as the primary treatment of FNFs continues to increase annually [13].

These changes in the application of the different surgical options depend on the results reported in several randomized trials and have been confirmed by meta-analyses that compared THA to hemiarthroplasty and internal fixation for displaced FNFs, showing that THA is associated with better outcomes and a lower incidence of re-operations [14,15].

Many factors can play a significant role in determining the outcome after THA for FNF. Recently, many studies have investigated the consequence of a surgical approach (direct anterior [DA], posterolateral [PL], and lateral direct [LD]) for total hip arthroplasty on short- and long-term follow-ups. Reported benefits of the DA approach are as follows: minor dislocation risk [16,17,18], lesser pain, and higher functional recovery in the early postoperative days (first 2 weeks after surgery) compared to the PL approach [19,20] and higher self-reported scores for functional recovery at 2 weeks after surgery compared to the LD approach [21]. However, these postoperative advantages often were absent at 3-month and 1-year follow-up evaluations [20,22].

Functional status in the first postoperative days after THA may play a role of higher importance in FNF patients considering their generally increased age and reduced level of activity compared to their elective counterparts [23].

Furthermore, growing evidence has suggested that early postoperative deambulation in the group of patients who suffered an FNF could influence mortality at follow-up [24,25,26,27,28].

The aim of this study is to identify the factors that lead to better outcomes in the early period and at 1-year follow-up in patients who underwent a THA operation after FNF.

## 2. Materials and Methods

### 2.1. Study Design and Patients

All patients who underwent cementless total hip arthroplasty for FNF from 1 January 2018 to 31 December 2022 were preliminarily identified. Patients who underwent THA for pathologic fracture, osteonecrosis, or lateral femoral fractures (trochanteric fractures) were excluded. Additionally, we considered exclusion criteria cases of bilateral FNF and the presence of ipsilateral acetabular injuries. Finally, patients aged more than 80 years old and subjects with a reported non-independent deambulatory capability at baseline were excluded.

The study was regularly approved by the North Aemilia Ethical Committee with number 631/2023 on 12 December 2023.

All procedures were performed after collecting written informed consent from each patient and in accordance with the ethical standards of the institutional and national committees of research and the 1964 Declaration of Helsinki and its subsequent amendments.

Patient data and demographic characteristics were collected, including age, gender, BMI, Garden classification of fracture, and ASA classification. Furthermore, we registered the following operative data: time between trauma and surgery (days), surgical approach (DA or LD), operative time, reported intraoperative complications (periprosthetic femoral fracture or surgical acetabular protrusion), and anesthesia type (spinal or general).

We verified, as previously reported, if the surgical procedure was performed by an arthroplasty-trained surgeon (AR-trained) or by a non-arthroplasty-trained surgeon (non-AR-trained).

### 2.2. Surgical Technique

In all patients, the THA was completed through an LD approach or a DA approach under spinal or general anesthesia. Both surgical approaches were performed in the supine position. 

For DA, a regular operating room table was used, and the patient was positioned with the hip over the table break junction, thus allowing table reflexion and hyperextension of the hip joint. The contralateral leg was typically inserted in the sterilization procedure before incision in order to make possible a correct check of limb length during surgery and a correct figure-four adduction during the femoral exposure. In obese patients, we conducted a pannus retraction with adhesive tape to avoid any possible related difficulties during stem broaching and positioning. An oblique incision was made originating 2–4 cm distal and lateral to the ASIS. Dissection was taken deep to expose the overlying thin fascia of the tensor fascia lata, which was then incised with the same axis as the cutaneous incision. Blunt finger dissection was utilized afterward, and the interval was developed between the sartorius and the TFL. Two wound protectors were positioned at this point in order to reach the minimization of soft tissue, muscle, and skin damage caused by retraction. The ascending branch of the lateral circumflex femoral artery and vein was identified and tied before cauterization, and the anterior hip capsule was exposed. The femoral neck osteotomy was then completed either with a single cut or sometimes with a napkin-ring-type parallel two-cut technique to facilitate the removal of the femoral head. Femoral exposure on our regular operating room table started with external rotation of the femur typically over 90°. A proximal femoral hook was useful in this phase to elevate the proximal femur and facilitate better exposure. The leg was extended by lowering the leg to the floor followed by adduction of the extremity. The femoral exposure was accomplished by reflexing the table to extend the extremity and placing the leg figure-four under the opposite leg with flexion of the knee of the operated leg lower to 45°. A retractor was then placed on the femur medial to the neck cut and a double-pronged retractor was placed under the greater trochanter. Soft tissue releases were performed identifying the piriformis fossa and proceeding along the greater trochanter while elevating the proximal femur with the hook device. The visualization of the osteotomy plane enabled the broaching and placement of a femoral trial component to commence. Closure of the wound was initiated with repair of the TFL fascia with either a running or interrupted suture. In all cases, a drain was correctly positioned.

For the direct lateral approach, we proceeded with releases of the anterior third of the gluteus medius and minimus while preserving the posterior femoral attachment of the major part of these muscles. The proximal part of the incision was limited by the superior gluteal nerve and vessels, crossing 3–5 cm proximal to the tip of the greater trochanter. Distally, the anterior fibers of the vastus lateralis were elevated from the anterior femur. Next, the anterior attachment of the hip capsule was released from the anterior base of the femoral neck, and an anterior longitudinal capsulotomy was opened as necessary with a proximal transverse T-shaped incision. Femoral exposure was completed with careful release of the postero-lateral capsule and positioning of the leg in figure-four under the opposite leg with a flexion of the knee over 90°. Closure of the wound started from the muscular fibers of the gluteus medius and minimus and then with fascia repair with an interrupted suture. In all cases, a drain was correctly positioned.

During all surgeries, intraoperative fluoroscopic imaging was used in order to exclude possible complications. The DA approaches were performed by two different surgeons, whereas the LD approaches were performed by five different surgeons. The arthroplasty-trained surgeons were the two surgeons able to implant THA with both the LD and DA approaches.

Perioperative and postoperative institutional protocols were adhered to with minor variations depending on surgeon preference. All patients received preoperative antibiotics before incision. 

If no surgical contraindications were present, then, in all patients, walking in the first 2 postoperative days was attempted with dedicated physiotherapy personnel. All included patients have a minimum of 1-year follow-up.

### 2.3. Radiographic Evaluation

Postoperative X-rays were checked by two orthopedic surgeons in order to verify correct component positioning: acetabular shell inclination and anteversion, as well as femoral subsidence and varus, were measured as previously reported in the literature [29,30].

Rotation of the proximal femur was verified by measuring the width of the lesser trochanter. Further fine adjustments were made using the ratio of the projection of the distal tip of the stem that was verified by known stem size. The inclination of the acetabular component referenced the interteardrop line and anteversion, following the previously reported method published by Haddad et al. [31]. The valgus/varus alignment of the stem was recorded using the method described by Khalily and Lester [32]. Subsidence was evaluated from the immediate postoperative radiographs and those at final follow-up as a vertical movement of the femoral component, as validated in previous study. 

The postoperative radiographs from the first ten patients were separately analyzed and evaluated by two independent observers to assess interobserver reliability through the single measures and with a two-way mixed effect intra-class correlation coefficient. The registered inter-observer reliability coefficient was in the correct range of tolerance for each radiographic assessment.

### 2.4. Outcome Measurement

In order to search for any predictive factors of outcome in the early period and at 1-year follow-up, we performed different multivariable logistic regression analyses.

Early outcome was evaluated using two different parameters: early deambulation (ED) and capability to reach autonomy in deambulation (AD) without crutches in the first month after surgical procedure.

ED was attempted in all included cases where the patients in the first 2 postoperative days were able to complete a supported deambulation of at least 20 meters. The results of this attempt were independently registered by four physiotherapists blinded to the design and scope of the study.

The level of autonomy in deambulation (AD) was evaluated by an orthopedic surgeon during one-month follow-up visit registering whether it was possible for deambulation without crutches and with a correct muscular response to weightbearing. The results in terms of AD were independently registered by surgeons blinded to the design and the scope of the study. 

Outcome at 1-year follow-up was evaluated using two different parameters: 1-year mortality and 1-year revision for any cause.

### 2.5. Statistical Analysis

All variables were checked in order to verify distribution, mean, median, interquartile range, and standard deviation.

A preliminary Kolmogorov–Smirnov test was performed for all variables in order to verify the normality distribution of the variable.

The variance inflation factor was used in order to verify the absence of multicollinearity inside each multivariate analysis.

The insertion of any variable in the multivariate analysis was followed by calculation of the derived adjusted R-squared to proceed to a correct adjustment of the models. 

A multivariable logistic regression analysis was performed for the two short-term outcome parameters including the data reported above. These two multivariate analyses were performed in order to identify predictive factors for failure in achieving early deambulation and an inability to reach deambulation without crutches at one-month follow-up.

A multivariable logistic regression analysis was then performed searching predictive factors for 1-year mortality and 1-year revision for any cause including the data reported above and including measurements of short-term outcomes.

The significance level was set at *p* < 0.05. 

Data elaboration and statistical analysis were performed using SPSS^®^ statistics software 20.0 (IBM^®^, Armonk, New York, NY, USA).

## 3. Results

**Figure 1:**
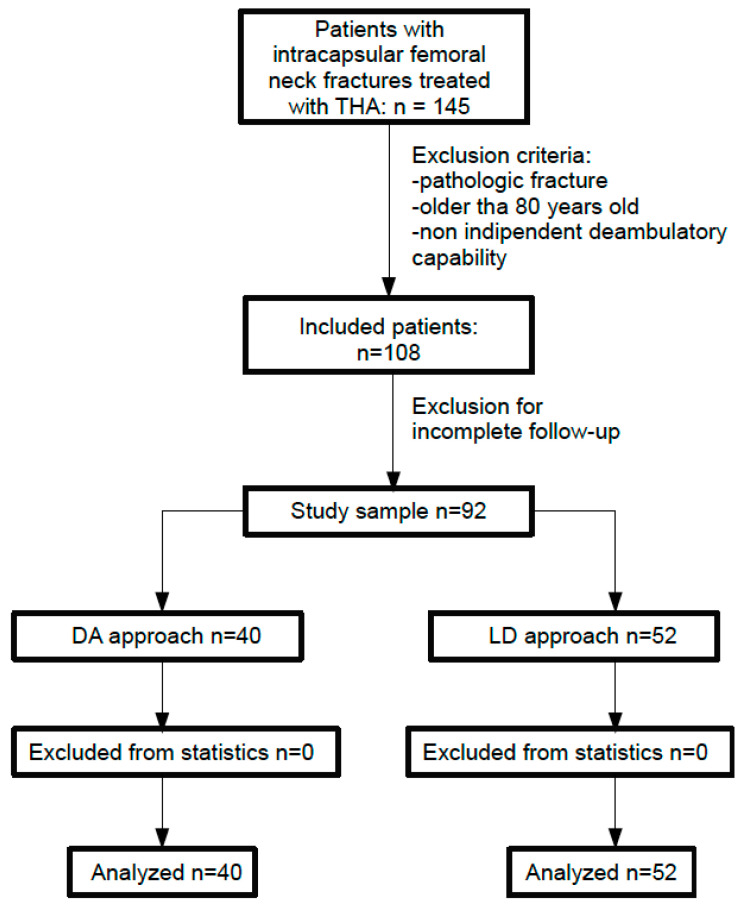
Flowchart of the studied population.

A total of 108 patients were preliminarily included in the study. After the final collection of data at follow-up, a total of 16 patients were excluded for unavailable complete information (Figure 1).

Thus, 92 patients constituted the pool of analysis of our study.

Demographic data are reported in Table 1.

An ED was registered as successful in 31 cases, and in 25 cases a successful AD at one month without crutches was possible with adequate muscular response to weightbearing.

The multivariable analysis for short-term outcomes is reported in Table 2 and Table 3.

**Figure 2:**
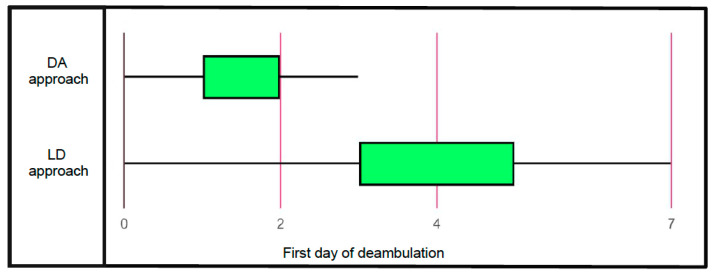
Graphical distribution of the first day of deambulation in Patients treated with direct anterior approach and lateral direct approach.

The logistic regression models showed the statistically significant predictive power of the surgical approach to influence ED. Specifically, the DA surgical approach could be considered a parameter able to predict the capability of reaching ED from our data (Figure 2). The second analysis performed for AD at one month showed the statistically significant predictive power of the surgical approach and ASA classification. Thus, the DA surgical approach and ASA classification < 3 were able to have a favorable influence on AD at one month.

The multivariable analysis for outcome at 1-year follow-up is reported in Table 4 and Table 5.

**Figure 3:**
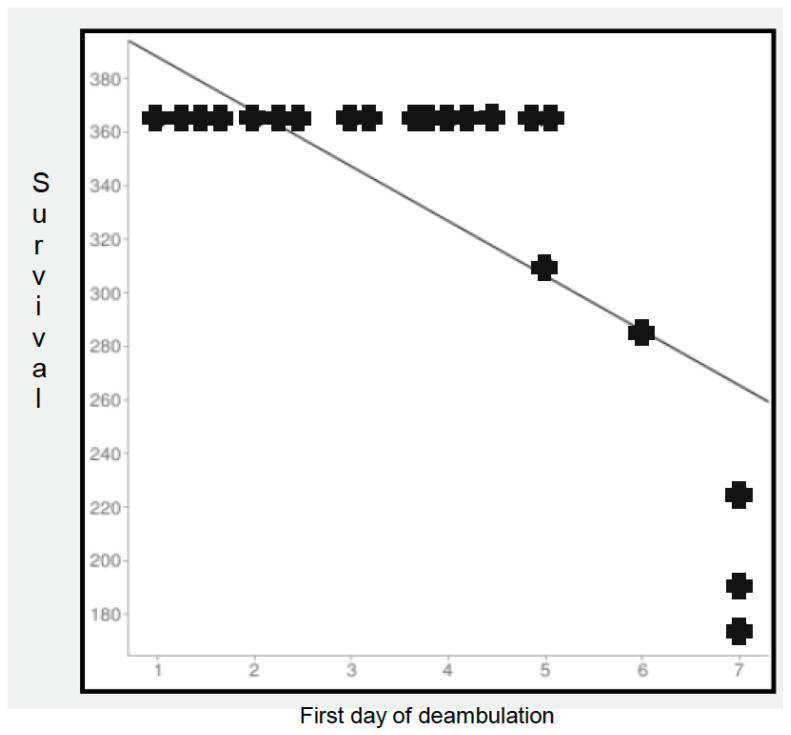
Survival of patients in relation to first day of registered deambulation.

The analysis for 1-year mortality identified statistical significance in the ASA classification. In particular, we documented a higher mortality risk at 1 year for ASA classification > 3. Furthermore, we considered, of particular interest, the strong near-significance value of ED in relation to 1-year mortality; in fact, even if it were not really significant, the value of ED in this model could witness the existence of the relationship between early mobilization and better survival in our data, as already reported in the literature (Figure 3).

Revision surgery was performed in four cases: three cases for atraumatic dislocation, and one case for early periprosthetic joint infection.

The analysis for 1-year revision risk showed statistical significance for the training of the surgeons. This logistic regression analysis documented a higher risk for revision when the surgical procedure was performed by a non-AR-trained surgeon.

## 4. Discussion

In this study, we present a case series of patients treated with THA after femoral neck fracture. After statistical processing of the data, we performed multivariate analyses in order to identify predictive factors of better outcomes in the first month and at 1-year follow-up.

From our data, it emerged that patients treated with THA through the DA surgical approach have a higher probability of reaching deambulation in the first days after surgery and to walk without crutches at one month. This could be derived from the minor muscular damage of this surgical approach, as is widely recognized in the literature. 

Furthermore, we performed a multivariate analysis for revision risk at one year, which documented a higher risk of re-operation for patients operated on by a surgeon without specific arthroplasty training.

In our opinion, it is also of particular interest that the near-significance value resulted in ED predicting 1-year mortality. We suggest further studies to investigate this issue in this specific population of fractured patients.

Fractures of the femoral neck have important economic implications due to their direct and indirect medical costs. Total hip arthroplasty is a surgical procedure with a documented increase in number and extension year-by-year for clinical and epidemiological reasons. A recent study of the National Database documented, in the last 22 years (from 2001 to 2022), a growth rate of hip replacement surgery of +8.19% [33]. In the same study, the increase in THAs remains significant, especially when considering the progressive aging of the population in the last 22 years [33]. A previous study published by Pabinger and Geissler [34] similarly reported that hip arthroplasty is increasing exponentially in the OECD (Organization for Economic Cooperation and Development) with different rates across the countries: USA, +12.87%; Australia, +7.77%; United Kingdom, +6.95%; Germany, +6.07%; and Spain, +6.73%.

There is growing evidence that total hip arthroplasty may result in improved outcomes over hemiarthroplasty and internal fixation [13,14]. Furthermore, recent studies have also reported that THA could be more cost-effective in these patients when compared to other surgical treatments [13,14].

Recently, some authors have demonstrated that hospitals in which there is management of a high proportion of hip fracture patients may be unfairly penalized with the current models of payment [35,36,37]. In fact, it is recognized that there are pronounced differences between patients who underwent THA for osteoarthritis and those for hip fractures. Patients with an FNF tend to be older and with more significant clinical associated problems, frequently experience an increased length of hospitalization, more often need to be discharged to a rehabilitation facility, and show a higher readmission rate after surgery [35,36,37].

In our study, we performed an analysis that demonstrated a better short-term outcome in patients who underwent THA with the DA surgical approach and a better long-term outcome in terms of 1-year revision in patients operated on by an AR-trained surgeon. Furthermore, we consider the influence of ED on 1-year mortality, which could be desumed from the logistic regression analysis, to be of particular interest. In our opinion, this relationship needs to be clarified in a larger cohort of patients.

While the surgical approach for THA in elective cases is still a topic of debate with recognized difficulties in comparing the large number of reported papers [38,39], few studies to date have compared different surgical approaches in performing THA after FNF [40,41]. The results of a recent study by Cichos et al. showed that THA performed with the DA approach is associated with a lower risk of dislocation and mechanical revision at both 3-month and 1-year follow-ups when compared to the PL approach. Similarly, those authors reported that the DA approach was associated with an improvement in mortality rates compared to the PL approach at both 3-month and 1-year registered follow-ups [41].

Furthermore, the same study reported that utilization of the DA approach determined a higher overall survival at follow-up when compared to other surgical approaches [41].

In the first systematic review and meta-analysis investigating surgical approaches for arthroplasty after FNF [42], nine eligible studies were included with publication dates between 2012 and 2016. Data from this meta-analysis showed that the DA approach provided better early functional mobility in four studies according to other data reported in the literature.

Bucs et al., in a study that evaluated the efficacy of the DA approach in HHA for FNFs compared to the DL approach, demonstrated that patients operated on with the DA approach reported less postoperative pain, resulting in patients more frequently being able to complete earlier mobilization [43].

Nogler et al. confirmed these data and showed in a different study that patients operated on with the DA approach had less postoperative pain, minor blood loss, and an inferior length of hospital stay when compared to those treated with the PL or DL approaches [44].

Multiple prior studies on elective total hip arthroplasty showed an improvement in early functional scores, deambulation, and mobilization in DA-approach patients for the first few weeks after surgery [18,19]. While the consequences of these early improved capabilities in mobilization and deambulation for long-term outcomes in elective patients have still not yet been completely clarified, the FNF population is generally in a worse position compared to their elective counterparts [22,36], given the potential major benefits of these early small differences. A previous study reported that the level of deambulation at 2 weeks after surgery could be considered as a significant predictor of survivorship at 1 year in the FNF population [23]. Therefore, in our opinion, we have to give particular importance to reaching early deambulation after THA for FNFs, and surgery through the DA approach has a demonstrated positive influence in achieving this result.

Several studies have recently investigated the role of surgeon training in the treatment of FNF. Padilla et al. recently reported an interesting study in which the direct medical costs of THA performed for FNFs between orthopedic surgeons trained in different subspecialties were compared [45]. These authors demonstrated that in the FNF population, surgeons with specific training in arthroplasty achieve lower total costs for the THA episode of care, whereas surgeons without specific training in arthroplasty often exceed the bundled payment target. Similar results were also reported by Thomas et al. [46].

Prior studies have mainly sought to elucidate the relationship between the volume and outcome of the surgical procedures, and have demonstrated that an increase in the number of patients operated on leads to an improved outcome [47,48,49,50]. Maceroli et al. [50] analyzed the New York State System database to determine if patient outcomes following THA performed for FNF differed between hospitals with a significant volume of completed procedures. Those authors reported that patients treated with total hip arthroplasty at the highest volume hospitals had significantly lower 30-day and 1-year mortality rates and a minor complication rate at 3-month follow-up.

Browne et al. [49], in their analysis of a nationwide database, identified 97,894 patients affected by FNF surgically treated in different hospitals with different surgeon volumes. They found that in-hospital mortality and complication rates were significantly higher in subjects treated by lower-volume surgeons than higher-volume surgeons, and that complication rates were higher in hospitals with a lower volume of performed procedures. We have to consider that their study cohort included patients who underwent internal fixation, hemiarthroplasty, and total hip arthroplasty, potentially limiting the generalizability of the results to a THA-only population.

Few studies have investigated the relationship between surgeon specialization training and outcomes [51,52]. Previous articles have encompassed a limited number of surgical specialties, and few have been published investigating this item in Orthopedics and Traumatology. Hagen et al. [53] identified patients who underwent primary or revision hip and knee arthroplasty in the United States to study the relationship between hospital specialization and outcomes. They found an inversely proportional relationship between a hospital’s degree of orthopedic specialization and the rates of adverse outcomes.

In fact, with an increase in orthopedic specialization, Hagen et al. [53] reported a progressive decrease in mortality, deep vein thrombosis, postoperative hemorrhage, and infection.

The results of our analysis on THA performed after FNF appear to be in accordance with the previous literature, underlining the importance of a surgical approach in determining a better outcome in the short term, leading to a better long-term outcome. Furthermore, our data confirm, as reported by other authors, that surgery for THA after FNF has significant advantages when performed by arthroplasty-trained surgeons.

This study should be interpreted considering its limitations. First of all, it has a retrospective design with the potential for transfer bias and the absence of randomization for surgical approaches. Additionally, the short- and long-term results at follow-up were collected by four different physiotherapists and by two different orthopedic surgeons. We followed previously published methods to verify correct femoral and acetabular positioning; however, all evaluations were performed by X-ray and not by CT imaging, with possible bias of underestimation. All people were blinded to the design of the study; however, possible bias could derive from excessive heterogeneity in participants in the data collection. Finally, this analysis was conducted on a limited number of patients at a single center and, thus, must be considered as only a preliminary indication to guide future multicenter prospective trials in order to reach definitive conclusions.

## 5. Conclusions

In our analysis, a direct anterior surgical approach and an ASA classification < 3 can predict improved results in the first month after THA for FNF. Furthermore, our results suggest that THAs performed by AR-trained surgeons have a lower probability of revision at 1 year. Mortality at 1 year was influenced by the ASA classification.

However, due to the results of our logistic regression models, we consider that the influence of early deambulation on 1-year mortality after THA for FNF is deserving of more clinical study.

## Figures and Tables

**Table 1:** Patient characteristics and demographic data.

	N = 92
Age (mean ± std dev)	72.6 ± 7.3
Gender	
Male	38
Female	54
BMI	25.8 ± 3.1
ASA	
1	8
2	29
3	51
4	4
Garden fracture	
2	5
3	56
4	31
Time trauma/surgery (hours)	57.1 ± 13.5
Operative time (min)	118 ± 14.9
Intraoperative complications	
Surg acetabular protrusion	4
Femoral fracture	2
Surgical approach	
DA	40
LD	52
Surgical training	
AR	43
non-AR	49
Anesthesia type	
General	34
Spinal	58
Component positioning	
Correct	75
Incorrect	
Stem	
Varus	3
Acetabular	
Inclination	7
Anteversion	5

**Table 2:** Multivariable logistic regression analysis for failure in early deambulation.

	OR	95% Confidence Interval	*p*
Age	0.781	0.314–0.827	0.083
Female	1.026	0.526–1.331	0.645
BMI	0.890	0.673–0.995	0.286
ASA < 3	0.762	0.540–0.913	0.109
Time trauma/surgery	1.153	0.732–1.385	0.622
Operative time	0.807	0.691–1.469	0.581
No intraoperative complications	0.996	0.503–1.542	0.875
DA surgical approach	0.291	0.106–0.374	**0.026**
AR-trained surgeon	0.467	0.238–0.782	0.171
Spinal anesthesia	0.957	0.580–1.463	0.602
Correct component positioning	0.554	0.296–0.831	0.185

DA: direct anterior; AR: arthroplasty. Bolded and underlined values identify variable with statistical significance.

**Table 3:** Multivariable logistic regression analysis for inability of deambulation without crutches at one-month follow-up.

	OR	95% Confidence Interval	*p*
Age	0.862	0.764–1.351	0.098
Female	0.615	0.391–1.227	0.189
BMI	0.928	0.732–1.416	0.352
ASA < 3	0.349	0.206–0.518	**0.034**
Time trauma/surgery	0.786	0.365–1.191	0.432
Operative time	0.883	0.475–1.364	0.502
No intraoperative complications	0.512	0.256–0.729	0.091
DA surgical approach	0.447	0.315–0.580	**0.026**
AR-trained surgeon	0.546	0.129–0.866	0.312
Spinal anesthesia	0.904	0.563–1.672	0.633
Correct component positioning	0.735	0.306–1.147	0.567

DA: direct anterior; AR: arthroplasty. Bolded and underlined values identify variable with statistical significance.

**Table 4:** Multivariable logistic regression analysis for revision surgery at one-year follow-up.

	OR	95% Confidence Interval	*p*
Age	0.741	0.489–1.266	0.659
Female	0.562	0.273–0.984	0.416
BMI	0.397	0.137–0.558	0.083
ASA < 3	0.604	0.218–1.165	0.687
Time trauma/surgery	0.856	0.649–1.453	0.705
Operative time	0.915	0.807–1.511	0.842
No intraoperative complications	0.789	0.625–1.139	0.821
DA surgical approach	0.423	0.156–0.727	0.234
AR-trained surgeon	0.258	0.114–0.392	**0.003**
Spinal anesthesia	0.891	0.565–1.376	0.908
Correct component positioning	0.672	0.496–0.978	0.536
Early deambulation completed	0.218	0.129–0.564	0.095

DA: direct anterior; AR: arthroplasty. Bolded and underlined values identify variable with statistical significance.

**Table 5:** Multivariable logistic regression analysis for mortality at one-year follow-up.

	OR	95% Confidence Interval	*p*
Age	0.471	0.206–0.872	0.247
Female	0.692	0.491–0.958	0.535
BMI	0.543	0.387–0.824	0.394
ASA < 3	0.367	0.194–0.525	**0.021**
Time trauma/surgery	0.825	0.657–1.237	0.780
Operative time	0.904	0.722–1.369	0.829
No intraoperative complications	0.786	0.315–1.196	0.452
DA surgical approach	0.217	0.108–0.313	0.096
AR-trained surgeon	0.338	0.239–0.482	0.081
Spinal anesthesia	1.026	0.524–1.361	0.755
Correct component positioning	0.487	0.280–0.712	0.274
Early deambulation completed	0.259	0.148–0.306	0.053

DA: direct anterior; AR: arthroplasty. Bolded and underlined values identify variable with statistical significance.

## Data Availability

Data are contained within the article.
